# Effect of frozen gloves on chemotherapy-induced neurotoxicity in breast cancer patients: a systematic review and meta-analysis

**DOI:** 10.3389/fonc.2024.1366782

**Published:** 2024-06-06

**Authors:** Hongting Wu, Ying Jin, Jiaqing Song, Xiufei Gao

**Affiliations:** ^1^ First Clinical Medical College, Zhejiang Chinese Medical University, Hangzhou, Zhejiang, China; ^2^ The First Affiliated Hospital of Zhejiang Chinese Medical University, Hangzhou, Zhejiang, China

**Keywords:** breast cancer, CIPN, cooling gloves, chemotherapy, peripheral neurotoxicity

## Abstract

**Background:**

Chemotherapy-induced peripheral neurotoxicity (CIPN) is a dose-limiting side effect observed in breast cancer patients. Its primary clinical manifestations include limb numbness, tingling sensations, hypoesthesia, or paresthesia. In severe instances, some patients may also encounter muscle cramps, weakness, and pain, leading to potential paralysis. The onset of CIPN significantly impacts the quality of life for cancer patients. Hence, it is imperative to explore preventive strategies for managing CIPN.

**Methods:**

We searched for relevant randomized controlled trials (RCTs) and non-randomized controlled trials (non-RCTs) in several databases. The primary outcome measures encompassed the Patient Neurotoxicity Questionnaire (PNQ), the Functional Assessment of Cancer Therapy-Taxane (FACT-Taxane), and the National Cancer Institute Common Terminology Criteria for Adverse Events (NCI-CTCAE). Secondary outcomes aimed to evaluate the quality of life and the tolerability of ice gloves. Meta-analysis was conducted using RevMan 5.3 software to determine the relative risk ratio (RR) and 95% confidence interval (CI).

**Results:**

We conducted an analysis involving 372 patients across seven trials. In our meta-analysis, the use of ice gloves demonstrated non-significant results in reducing the incidence of both motor and sensory neuropathy, as assessed through CTCAE (sensory: RR: 0.94; 95% CI: 0.85 to 1.02; P = 0.15; motor: RR: 1.04; 95% CI: 0.88 to 1.22; P = 0.64). Similarly, when evaluated using the PNQ, there was no significant reduction observed in the incidence of sensory and motor neuropathy (sensory: RR: 0.49; 95% CI: 0.20 to 1.20; P = 0.12; motor: RR: 0.71; 95% CI: 0.26 to 1.99; P = 0.52). Consistently, our conclusions remained unchanged when employing the FACT-Taxane assessment. Regarding the evaluation of the quality of life, our observations suggested a potential improvement with the use of ice gloves, and participants exhibited moderate tolerance towards them.

**Conclusion:**

Ice gloves are a reasonable option for the treatment of CIPN in patients undergoing chemotherapy for breast cancer. However, the effectiveness of ice gloves in combating CIPN remains inconclusive at this time due to the low quality and limited number of clinical trials on this topic.

**Systematic review registration:**

https://www.crd.york.ac.uk/prospero/display_record.php?ID=CRD42023457045, identifier CRD42023457045.

## 1 Introduction

In women, breast cancer is the most common cancer and the leading cause of cancer death (1). Chemotherapy has gained global recognition as a pivotal and indispensable treatment for breast cancer. However, while targeting tumor cells, chemotherapy drugs frequently inflict damage on normal cells indiscriminately. The resulting sensory impairment due to damage to peripheral or autonomic nerves is termed ‘Chemotherapy-induced peripheral neurotoxicity (CIPN)’ (2).

Chemotherapy-induced peripheral neurotoxicity (CIPN) serves as a dose-limiting side effect commonly associated with various classes of chemotherapy drugs. These include platinum compounds, periwinkle alkaloids, taxanes, proteasome inhibitors, and immunomodulators (3). Notably, among these, platinum drugs and taxanes stand out as the most neurotoxic categories (2). The incidence of CIPN ranges broadly from 30% to 90%, contingent on the specific neurotoxic drug, cumulative dosage, and pre-existing neuropathic conditions (4). The primary clinical manifestations of CIPN encompass limb numbness, tingling sensations, hypoesthesia or paresthesia, accompanied by muscle spasms, weakness, pain, and, in severe cases, potential paralysis (5). Early-onset CIPN often restricts a patient’s mobility, hampering recovery in the affected limb. As survival extends, adverse symptoms tend to exacerbate gradually. The persistence of CIPN over the long term, or even permanently, amplifies patients’ psychological burden and significantly impacts their quality of life (6). Approximately 40% of patients experience complete symptom reversal within 4 to 6 months following treatment cessation. However, 10% to 30% may still develop CIPN several years after treatment discontinuation (7). The emergence of CIPN greatly affects the quality of life of cancer patients, so it is necessary to find a prevention and treatment method for CIPN.

Duloxetine emerges as a potential therapeutic option for alleviating symptoms, supported by phase III randomized controlled trials highlighted in the American Society of Clinical Oncology (ASCO) guidelines for managing CIPN. However, duloxetine is prone to adverse events, such as drowsiness and thirst, and is limited by low tolerance, so its use is not recommended. Currently, there are no specific first-line agents recommended for the treatment and prevention of CIPN (8). Non-pharmacological therapies primarily aim to repair damaged neuronal cells by enhancing microcirculation and nutrient metabolism around nerves. These encompass exercise therapy, physical therapy, acupuncture, massage, dietary adjustments, and cryotherapy (9). Among them, cryotherapy mainly includes cooling gloves and socks, limb-induced hypothermia or cryocompression, and crushed ice compresses (10–12). There have been several studies investigating the efficacy of cryotherapy in preventing chemotherapy-induced peripheral neuropathy (CIPN) (13, 14), and most studies believe that the preventive function of cryotherapy is to reduce the toxic delivery of chemotherapy by constricting blood vessels and reducing local perfusion, which ultimately alleviates the complications caused by chemotherapy and alleviates the discomfort of patients (15, 16). However, on the other hand, there is no literature on the therapeutic effect of cryotherapy, so we cannot conclude the therapeutic effect of cryotherapy on CIPN or explore its mechanism of action. This review specifically aims to examine the use of frozen gloves in cryotherapy, compiling and analyzing available evidence to assess the effects of cold frozen gloves on chemotherapy-induced peripheral neurotoxicity in breast cancer patients.

## 2 Methods

### 2.1 Protocol and registration

This systematic review and meta-analysis adhered to the Preferred Reporting Items for Systematic Review and Meta-Analysis (PRISMA) guidelines (17). The study’s protocol was registered in PROSPERO with the registration number CRD42023457045.

### 2.2 Search strategy

Database retrieval strategies were formulated according to the requirements of the Cochrane Systematic Review Manual, and computer retrieval was the main method. A comprehensive literature search was conducted in the Web of Science, PubMed, Cochrane Library, Embase, Scopus, EBSCO, OVID databases, the China National Knowledge Infrastructure database (CNKI), and the Wanfang Data Knowledge Service Platform. All the publications until August 7, 2023, were searched without any restriction of country or article type. The search terms were a combination of subject terms and free words and include terms such as “breast cancer,” “chemotherapy-induced neurotoxicity,” “frozen gloves,” and “cryotherapy.” Specific search strategies can be found in the **Supplementary Materials**.

To ensure a comprehensive article search, we extended our search to clinical trial registry websites, such as ClinicalTrials.gov, the International Clinical Trials Registry Platform (ICTRP), and grey literature websites (open grey. eu). Our search was limited to publications in English or Chinese.

### 2.3 Eligibility criteria

To start, we eliminated all duplicate references. Two independent researchers selected the relevant reviews by screening the titles and abstracts of the identified articles. The full texts of these were then retrieved for further assessment of their potential eligibility. In cases where a consensus was not reached, any disagreements regarding inclusion were resolved through discussion or by consulting a third evaluator.

The inclusion criteria comprised: (1) Research subjects: patients diagnosed with breast malignancy through pathology or histology, without CIPN symptoms. (2) Study types: randomized controlled trials and non-randomized controlled trials. (3) Intervention: The control group received a non-ice glove intervention, while the treatment group received an ice glove intervention. (4) Language: the included articles were in English or Chinese.

The exclusion criteria encompassed: (1) the unavailability of full text. (2) Article types including discussion papers, letters, reviews, conference reports, and other publications. (3) Presence of underlying diseases predisposing to CIPN. (4) Included articles where CIPN was not the primary outcome.

### 2.4 Data and outcome extraction

Two reviewers individually read articles and extract specific data points, including author details, publication year, trial location, participant count, participant age, received chemotherapy regimen, and outcome measures. Any discrepancies between reviewers during article reading and data extraction were resolved through consultation with a third reviewer. In cases of unavailable data, authors were promptly contacted via phone or email for the necessary information.

The predetermined primary outcome was the incidence and severity of CIPN, and evaluation metrics were: FACT-T, PNQ, and CTCAE. The secondary outcome was an assessment of quality of life and the patient’s tolerance to ice gloves.

The Patient Neurotoxicity Questionnaire (PNQ) (18) comprises two graded items—sensory and motor—based on the patient’s subjective responses, ranging from A (no neuropathy) to E (severe neuropathy). The boundary between class C and class D delineates the absence (≤ class C) or presence (≥ class D) of symptoms interfering with daily activities. Identification of affected activities is crucial for patients experiencing ≥D symptoms. The Functional Assessment of Cancer Therapy-Taxane (FACT-Taxane) (19) is comprised of the FACT-General (FACT-G) plus a 16-item Taxane subscale, in which the Taxane subscale combines 11 items on the neurotoxicity subscale and 5 additional questions assessing symptoms associated with arthralgia, myalgia, and skin discoloration. The scale uses reverse scoring: A lower score corresponds to a decrease in quality of life and an increase in CIPN frequency. The National Cancer Institute Common Terminology Criteria for Adverse Events (NCI-CTCAE) (20) combines sensory and motor assessment items into a unified scale, generating a total assessment score: Grade 0 = asymptomatic; Grade 1=asymptomatic, loss of deep tendon reflexes or paresthesia; Grade 2=moderate symptoms, limiting instrumental activities of daily living.

### 2.5 Risk of bias assessment

Two reviewers assessed the included studies. Any inconsistencies resolved through discussion or consultation with the third reviewer. Assessment of randomized controlled trials employed the Cochrane risk of bias tool (21), with each evaluated item categorized as low risk (+), high risk (–), or unclear ()?. Non-randomized controlled experiments were evaluated using the Methodological Index for Non-Randomized Studies (MINORS) (22), consisting of 12 evaluation indicators, each scored from 0 to 2 points. A maximum score of 24 points is possible. A score of 0 indicates that it is not reported, a score of 1 indicates that it is reported but with insufficient information, and a score of 2 indicates that it is reported and sufficient information is provided.

### 2.6 Statistical analysis

The meta-analysis used Review Manager software to analyze the data, the continuous data was analyzed using a standardized mean difference (SMD) with 95% confidence intervals (CI), and the dichotomous data were assessed using odds ratios (ORs) and 95% CI. We assessed heterogeneity using the I^2^ statistic and Q-test. A fixed-effect model was used when heterogeneity was not statistically significant (P > 0.10, I^2^ < 50%). Conversely, a random-effects model was used in cases of significant statistical heterogeneity (P ≤ 0.10, I^2^ > 50%). The final data was visually represented using a forest plot. When the observed metrics could not be combined, we used descriptive language to express the results.

## 3 Results

### 3.1 Study selection

**Figure 1:**
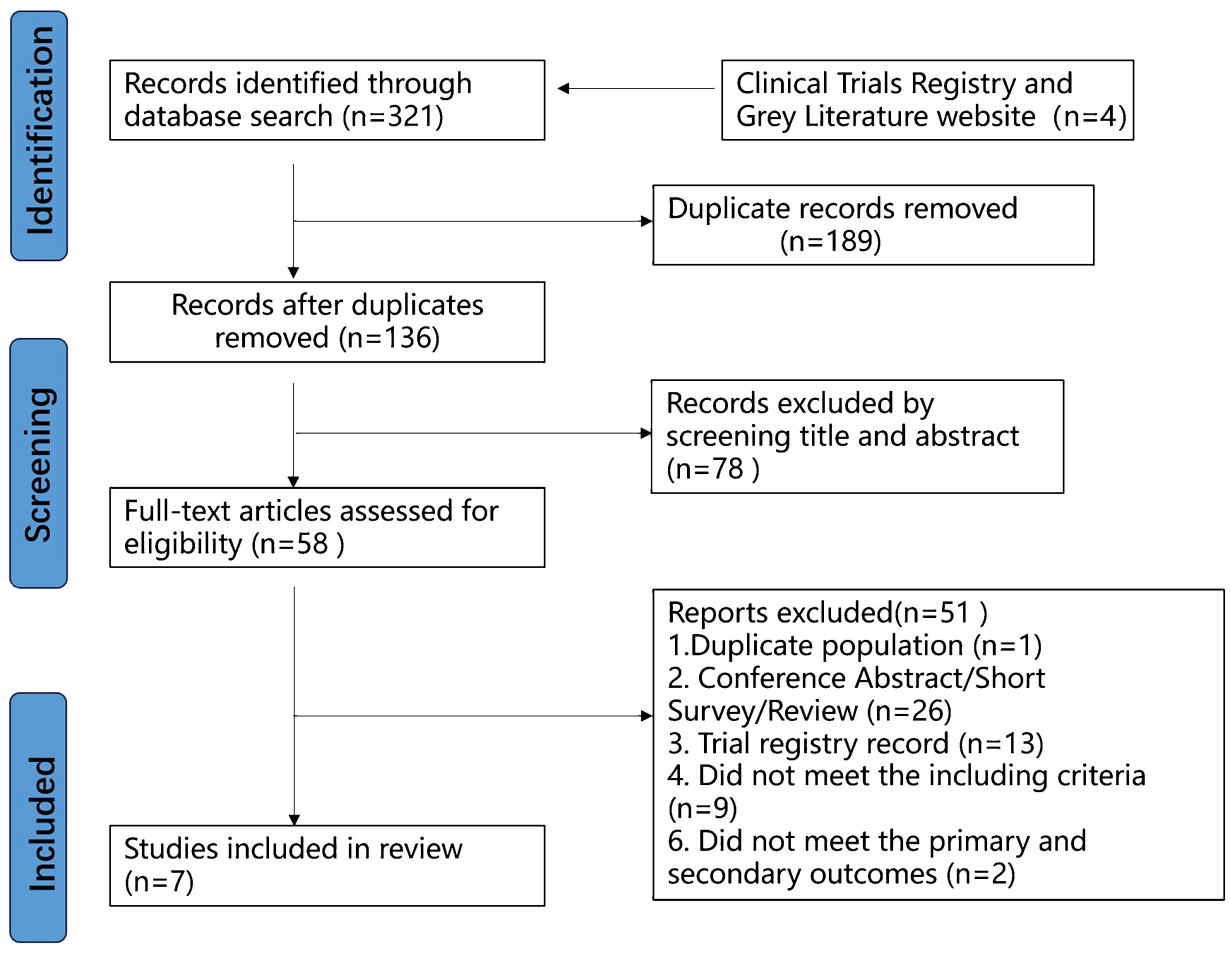
Flow chart of the study selection process.

**Figure 1** illustrates the flow chart outlining our search and study selection strategies. A total of 321 records were initially identified across multiple databases: Web of Science (n=44), PubMed (n=22), Cochrane Library (n=41), Embase (n=79), Scopus (n=79), EBSCO (n=18), OVID databases (n=37), China National Knowledge Infrastructure database (CNKI) (n=0), and Wanfang Data Knowledge Service Platform (n=1). Additionally, four results were sourced from clinical trial registry websites (n=4), while no relevant results were obtained from grey literature websites (n = 0). 189 duplicate records were identified and removed. Subsequently, 78 irrelevant records were excluded based on title and abstract screening. Upon full-text screening, 51 trials were excluded, of which 26 were reviews or conference abstracts. Furthermore, 13 experimental records lacked complete experimental results and were therefore excluded. One trial utilizing crushed ice rather than ice gloves (NCT02640053) and another involving gynecological cancer patients instead of breast cancer patients (TCTR20210129001) were both excluded. An article was written in Japanese and seven studies either unrelated to or not including ice gloves were also excluded. Additionally, three articles were excluded due to mismatched population and outcome measures. Moreover, two articles by Shigematsu with identical population and outcome measures led to the exclusion of the 2023 retrospective study (23) and the inclusion of the 2020 randomized controlled trial (24). Finally, 7 studies were included.

### 3.2 Study characteristics

**Table 1** shows the characteristics of all seven studies, including four RCT studies, one self-randomized controlled study, and two self-non-randomized controlled trials. All seven studies had a total of 372 participants. For primary outcomes, four studies reported PNQ, three trials reported FACT-Taxane and three studies reported CTCAE. In addition, for secondary outcomes, three studies assessed quality of life, and four studies referred to patient tolerance of ice gloves.

**Table 1:** The characteristics of the included trials.

Study	Country	Trial Type	Age (years)	Type of chemotherapy	Sample size	Ice gloves temperature	Ice gloves use time	Observe time	Outcome measures
**Mei-Ying,Jue(2022) (** 25)	America	randomized controlled trial	Mean: Control:47 Treatment:51	paclitaxel	n=48	(-24°C~-20°C)	75min	16 weeks	FACT-Taxane, CTCAE
**Hideo Shigematsu(2020) (** 24)	Japan	randomized phase II controlled trial	≥20	paclitaxel	n=44	(-20°C)	90min	12 weeks	FACT-NTX, PNQ, CTCAE, FACT-Taxane
**Ding Quan Ng(2020) (** 26)	Singapore	randomized controlled trial	Mean: Control:53.6 Treatment:56.4	paclitaxel	n=46	(-20°C~-10°C)	90min	9 months	PNQ, EORTC QLQ-CIPN 20, EORTC QLQ-C30, Electrophysiological assessments(NCS, SSR)
**Min Xu (2023) (** 27)	China	randomized controlled trial	Mean:49.93	nab-paclitaxel, paclitaxel, docetaxel, and carboplatin	n=129	(-10°C~4°C)	60min	7 days	CIPNAT, EORTC QLQ-C30, CTCAE
**Yuko Kanbayashi(2019**) (28)	Japan	self-randomized control study	Mean:57.6	Nab-paclitaxel	n=43	\	60min	12 weeks	CTCAE , PNQ, FACT-Taxane
**Akiko Hanai (2017**) (15)	Japan	self-controlled clinical trial	Mean:56	paclitaxel	n=40	\	90min	12 weeks	the incidence of CIPN (Objective Symptoms), PNQ(Subjective Symptoms)
**Ting-Ting Yang (2022**) (29)	China	self-controlled study	Mean:56.52	paclitaxel	n=22	(-24.3°C~-24.7°C)	90min	16 weeks	EORTC QLQ-CIPN20

### 3.3 Risk of bias

**Figure 2:**
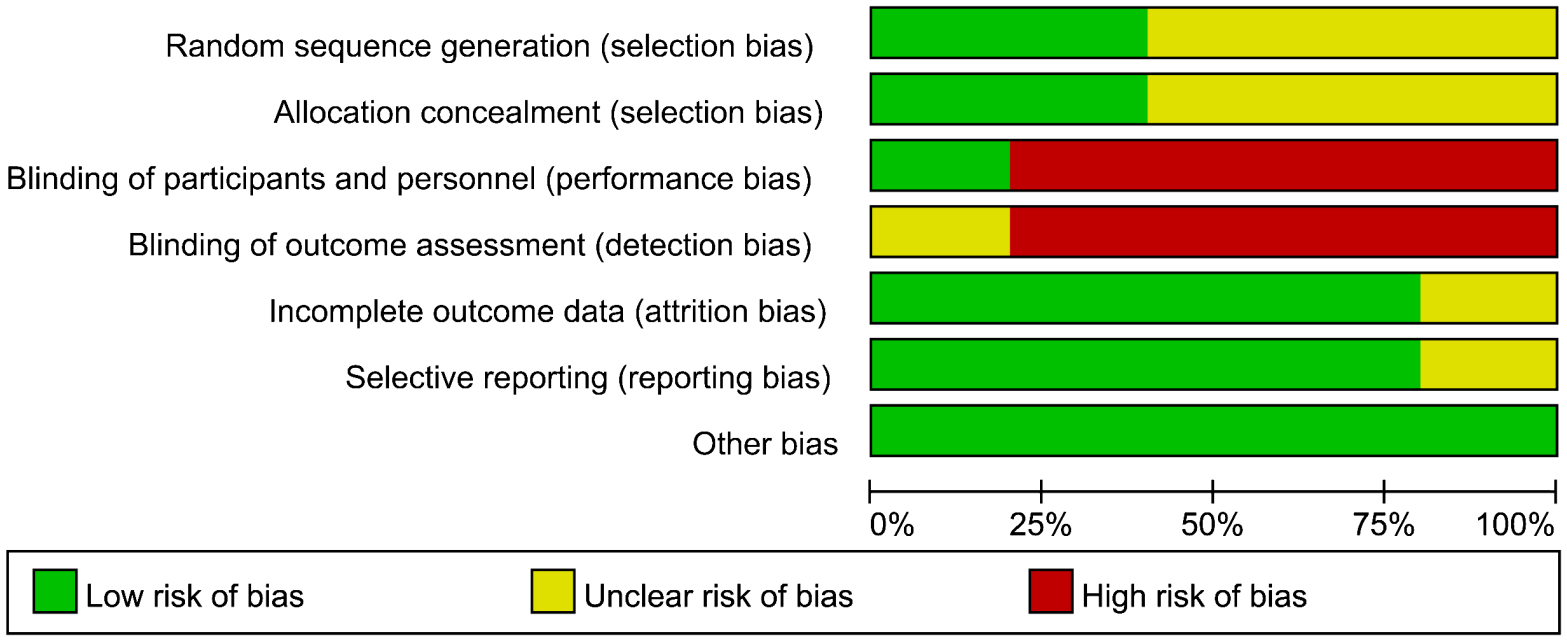
Risk of bias graph for RCT.

**Figure 3:**
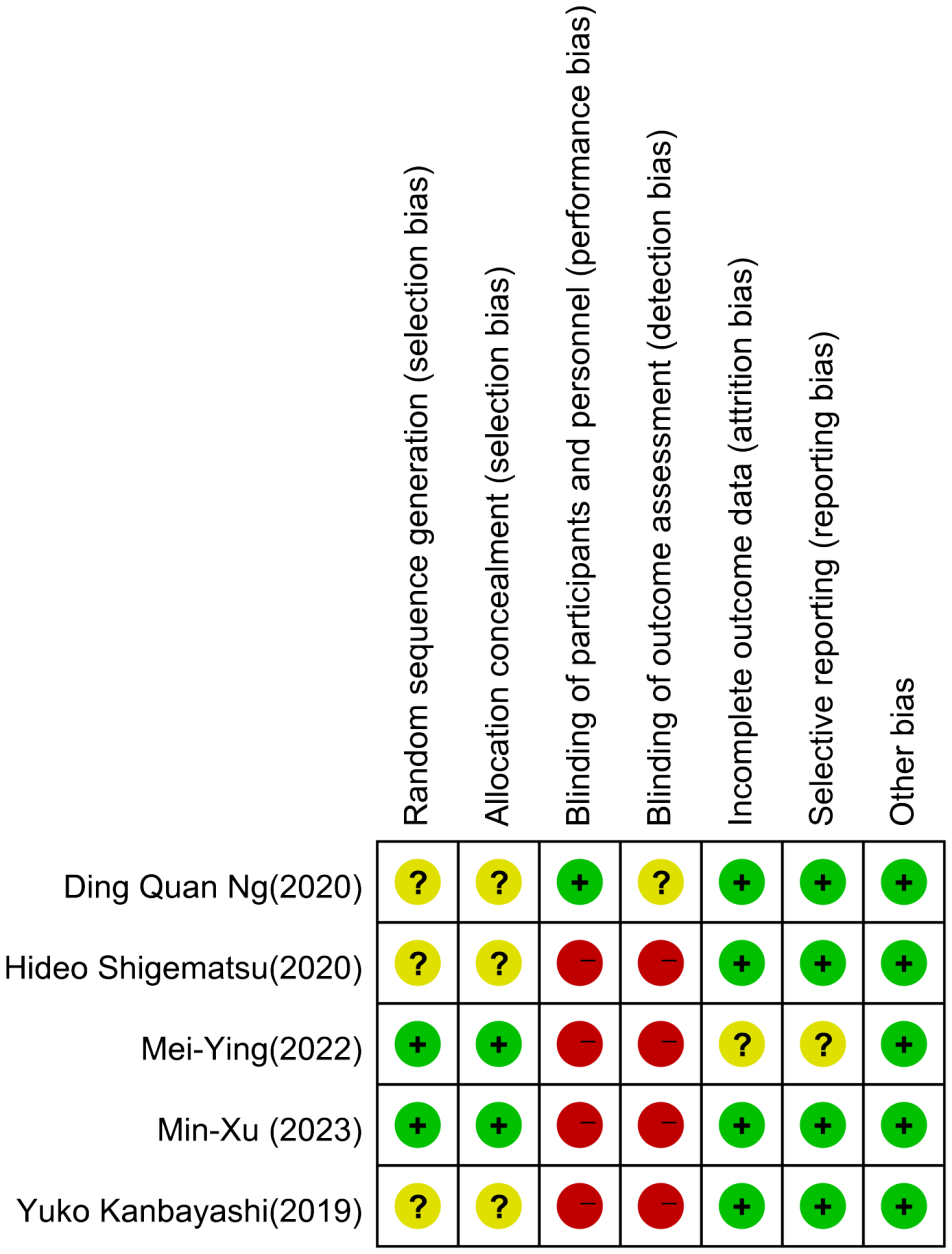
Risk of bias summary for RCT.

The risk of bias in RCT studies is shown in **Figures 2**, **3**. The majority of these studies were categorized as ‘high risk’ due to the absence of explicit blinding protocols. In assessing the quality of non-randomized controlled trials, we utilized the MINORS scale, as detailed in **Table 2**. The two non-RCT studies received scores of 16 and 17, respectively. Due to the limited number of articles included, we did not make funnel plots for publication bias analysis.

**Table 2:** Non-RCTs quality evaluation table.

	A clearly stated aim	Inclusion of consecutive patients	Prospective collection of data	Endpoints appropriate to the aim of the study	Unbiased assessment of the study endpoint	Follow-up period appropriate to the aim of the study	Loss to follow up less than 5%	Prospective calculation of the study size	An adequate control group	Contemporary groups	Baseline equivalence of groups	Adequate statistical analyses	Score
Akiko Hanai (2017) (15)	2	2	0	2	0	1	2	1	1	2	1	2	16
Ting-Ting Yang (2022) (29)	2	2	0	2	0	1	2	2	1	2	1	2	17

### 3.4 Outcomes

#### 3.4.1 Incidence of CIPN

**Figure 4:**
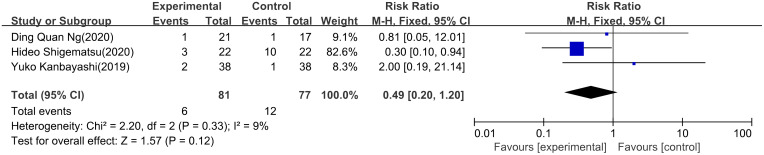
PNQ sensory.

**Figure 5:**
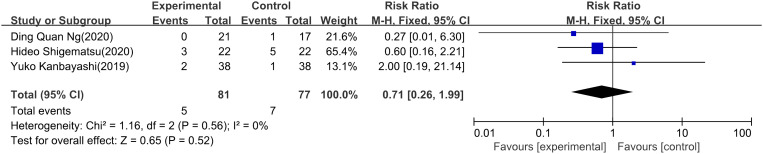
PNQ motor.

PNQ Among the four trials (3 RCTs and 1 non-RCT) reporting on PNQ scale usage, the event point of PNQ level ≥D was employed. Regarding PNQ sensory, a meta-analysis included 3 studies (24, 26, 28) revealed no statistically significant difference in the prevention and treatment of chemotherapy-induced neurotoxicity between the use of ice gloves (RR: 0.49; 95% CI: 0.20 to 1.20; P = 0.12). Additionally, there was no significant heterogeneity observed (I^2^ = 9%) (**Figure 4**). In the case of the PNQ motor, meta-analysis also showed no statistically significant disparity in the preventive and control effects of ice gloves (RR: 0.71; 95% CI: 0.26 to 1.99; P = 0.52), with no significant heterogeneity (I^2^ = 0%) (**Figure 5**).

Akiko Hanai’s (15) non-RCT study focused on sensory impairment, examining PNQ grades in both hands and feet. The analysis involved 36 participants, revealing the following outcomes: For two-hand PNQ≥D, the experimental group showed a rate of 2.8%, while the control group exhibited a significantly higher rate of 41.7% (OR = infinite, 95% CI = 3.32 to infinite, P < 0.001). Similarly, regarding two-foot PNQ≥D, the experimental group displayed a rate of 2.8%, contrasting with 36.1% in the control group (OR = infinite, 95% CI =2.78 to infinite, P<0.001). These findings highlight substantial differences in PNQ grades between the experimental and control groups in both hand and foot assessments.

FACT-Taxane In Kanbayashi’s study (28), a self-randomized controlled trial compared cryo-gloves and regular small surgical gloves, randomly assigning them to the dominant and non-dominant sides. The study found no significant difference in FACT-T total scores between the groups at assessment time (P = 0.67-0.93). Shigematsu’s research (24) demonstrated a respective reduction of 2.0 and 4.6 points in mean FACT-Taxane scores in the cryotherapy and control groups. The cryotherapy group exhibited a lower change (95% CI, 0.4 to 4.8; p = 0.02). The lower the score, the lower the quality of life, and the higher the frequency of PN, which means the ice glove treatment effect is better than that of the control group. Mei-Ying Jue (25) utilized the TaxS subscale with inverted scoring, where lower scores indicate stronger CIPN symptoms. Both groups, conventional treatment, and cold therapy, showed an increasing trend in CIPN frequency (score decreasing over time). However, the difference in trend between the groups was statistically significant (P < 0.05), with the frozen gloves group exhibiting significantly higher scores than the control group.

**Figure 6:**
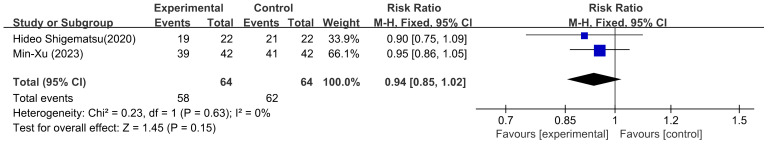
CTCAE sensory.

**Figure 7:**
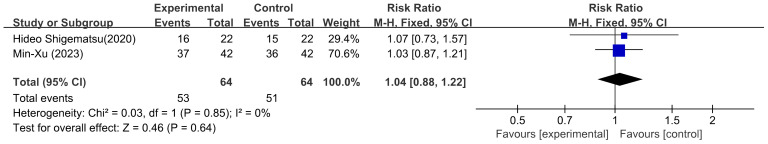
CTCAE motor.

CTCAE Mei-Ying Jue’s study (25) revealed that participants undergoing conventional treatment were approximately three times more likely to develop CIPN and progress to severe neuropathy compared to those receiving cold therapy (OR = 3.64, 95% CI = 2.22-5.97, p < 0.001). Moreover, a meta-analysis of two other RCTs (24, 27) indicated no statistically significant difference in the prevention and control of CIPN concerning sensory (RR: 0.94; 95% CI: 0.85 to 1.02; P = 0.15; **Figure 6**) or motor (RR: 1.04; 95% CI: 0.88 to 1.22; P = 0.64; **Figure 7**) aspects. There was no significant heterogeneity observed.

#### 3.4.2 Quality of life

Min Xu (27) and Ding Quan (26) administered a questionnaire based on the EORTC QLQ-C30 scale (30) for each patient. Min Xu’s study demonstrated significantly higher scores in various aspects for the experimental group compared to the control group. Most notably, physical function (85.87 vs. 82.76, P = 0.004) and overall quality of life (65.08 vs. 50.20, P < 0.001) exhibited significant differences. These findings suggest the potential benefits of cryotherapy for breast cancer patients undergoing chemotherapy in terms of their quality of life. However, a mixed-effects model analysis of GHS, PF, RF, and pain C30 subscale scores in Ding Quan’s trial (26) showed no difference between cryotherapy and control participants. Mei-Ying Jue (25) measured quality of life using the FACT-General (FACT-G) subscale, where higher scores indicate better quality of life. Their results showed no significant difference in quality of life between the two groups.

#### 3.4.3 Tolerance to ice gloves

In Shigematsu’s study (24), 32% of patients in the freezing group exhibited poor tolerance to ice gloves; however, no serious side effects were observed. Additionally, in Kanbayashi’s study (28), two patients withdrew from the trial due to an inability to tolerate cryotherapy. In Ding Quan’s trial (26), 80.9% (17/21) of participants required temporary interruption of cryotherapy at least once throughout chemotherapy due to cold intolerance, and no other serious adverse events secondary to cryotherapy were observed. Akiko Hanai (15) reported that no patients withdrew due to intolerance during the study.

## 4 Discussion

This review examines the preventive effects of frozen gloves on chemotherapy-induced peripheral neuropathy (CIPN) in women with breast cancer. It includes four randomized controlled studies, one self-randomized controlled study, and two non-randomised controlled studies, involving a total of 372 participants. The primary outcome measures were the incidence of CIPN, assessed using three commonly used scales: PNQ, FACT-Taxane, and CTCAE. Meta-analyses indicated that ice gloves did not have a clear preventive effect on the occurrence of CIPN. In the included studies, most participants wore frozen gloves or socks on their hands and feet for 90 minutes continuously at -20°C, starting 15 minutes before paclitaxel infusion and ending 15 minutes after infusion. The observation periods varied across the studies, ranging from 1 week to 9 months. The differences in observation time may have contributed to the variability in the experimental results. Only three trials provided clear information on the effects of ice gloves on patients’ quality of life, and the findings were inconclusive. Four trials reported on the tolerability of ice gloves, and it was noted that some patients experienced difficulty tolerating the low temperatures. However, researchers adapted the use of ice gloves over time to reduce the risk of severe frostbite from prolonged exposure to low temperatures.

Ice gloves, a form of cryotherapy, represent a safe and easily applicable method within clinical settings (31). A meta-analysis of cryotherapy for the prevention of TIPN in taxane patients (32) incorporated nine experiments utilizing cryotherapy techniques, such as frozen gloves and socks, whole-limb cryocompression, continuous-flow cooling, and regional cooling. Utilizing CTCAE as the primary outcome measure, the findings suggest an uncertain efficacy of cryotherapy in preventing CIPN. Similarly, a systematic review of 11 trials evaluating cryotherapy’s efficacy and safety for CIPN (33) revealed mixed results. Roughly half of the studies demonstrated significant improvement in at least one endpoint related to preventing CIPN. A multicenter randomized controlled trial (34) was conducted, involving patients with various types of cancer. The experimental group used ice gloves, while no significant improvement was observed in the chemotherapy-induced peripheral neuropathy (CIPN) subscale reported by patients. These findings are consistent with the results summarized in this study, indicating that ice gloves do not have a significant preventive effect on CIPN.

The pathological and physiological mechanisms of chemotherapy-induced peripheral neuropathy (CIPN) are currently unclear. Potential targets of platinum agents and taxane analogs include microtubules in the dorsal root ganglia, axonal components, ion channels, and the mitochondria of peripheral nerve fibers. These targets are believed to be involved in the development of nerve damage and related neuropathic symptoms in CIPN. However, further research is still needed to fully understand the complex mechanisms of CIPN (4). Some researchers believe that chemotherapy-induced peripheral neuropathy (CIPN) is dose-dependent and exhibits a length-dependent distribution, meaning that higher doses of chemotherapy drugs are more likely to cause nerve damage, with the nerves furthest from the spinal cord being the most affected. This hypothesis suggests that reduced delivery of chemotherapy drugs to the peripheral nerves may have a neuroprotective effect and potentially reduce nerve damage (34). For example, one animal study showed that cryotherapy prevents TIPN through local vasoconstriction, thereby reducing the delivery of neurotoxic chemotherapy drugs to peripheral nerves (35). Another *in vivo* study showed that local hypothermia reduced sciatic nerve blood flow and neurometabolism in rats (36), this suggests a reduction in the cumulative toxic chemotherapy dose near the distal nerve fibers, ultimately highlighting the role of ice gloves in the fight against CIPN. In the above example, cryotherapy is mainly used for the patient’s hands and/or feet. When the epidermal temperature drops to about 20°C, blood flow is reported to decrease by about 50% (37), A local temperature that is too low may reduce the drug distribution at the corresponding location. From a mechanical perspective, cryotherapy can reduce the distribution of taxanes on the hands and feet, further reducing the aggregation and binding of microtubules, inhibiting changes in cell shape and cell stability, and impairing axonal transport of essential cellular components, ultimately preventing the degeneration of distal nerve segments and axonal membrane remodeling (2). Chemotherapy drug-induced sensory nerve abnormalities such as pain, numbness, and tingling often occur in the hands and feet, Topical cryotherapy may directly affect sensory neuropathy. In summary, from the theoretical and related experimental analyses, it is concluded that cryotherapy can reduce the occurrence of neuropathy.

There is no clear statement about the preventive effect of ice gloves on CIPN. We speculate that one of the reasons may be related to chemotherapy regimens. The incidence and severity of CIPN are closely related to the type and dose of chemotherapy drugs used. Shigematsu (24) also mentioned that changes in chemotherapy regimens may lead to differences in the incidence of peripheral neuropathy. In this review, paclitaxel was used in all included trials, related research (4) has shown that these drugs cause the most neurotoxicity and are therefore likely to affect the preventive effect of ice gloves, so we cannot definitively deny that ice gloves do not have any effect on breast cancer CIPN. In addition to the specific protocol, the duration of use of ice gloves can also have a significant impact on the outcome of the intervention. Temperature data from Aishwarya (12) showed that the first 60 minutes are the period during which the ice gloves have the greatest cooling effect, after which the body tissue will reach thermal stability and vasoconstriction will also weaken, so achieving this state and generating sufficient vasoconstriction before chemotherapy starts is key to relieving CIPN. However, most of the trials included in this review started using ice gloves 15 minutes before chemotherapy, when the skin did not reach an optimal freezing state, which greatly affected vasoconstriction and reduced the effect of ice gloves on neurotoxicity. In addition, the effect of ice gloves is not persistent or unstable, requires replacement of frozen gloves every 45-60 minutes, lacks thermal homeostasis, and therefore may reduce the efficacy of vasoconstriction.

This systematic review and meta-analysis focused on the preventive effect of frozen gloves on neurotoxicity after chemotherapy in breast patients. Still, the small sample size of the studies included in this study was because we changed the intervention from a variety of cryotherapy to ice gloves alone. The lack of large randomized controlled trials of CIPN and ice gloves may bias the results. Secondly, the included trials were not explicitly blind, which may cause information bias. In addition, due to the limitation of the number of studies, this paper did not conduct bias analysis, which is an important methodological limitation, and the overall quality of the study is low. The quality of the evidence was limited by the small sample size. Third, some outcome measures include different types of experiments with insufficient data similarity, so the variability of the results is large, such as the PNQ scale evaluation results of DingQuan, Shigematsu, and Kanbayashi. The included studies had a short follow-up period, with a minimum follow-up of only seven days, which may also affect the assessment of the prognosis of CIPN. Therefore, we should be cautious in interpreting the results of the study.

In addition, for the assessment of CIPN, there is currently a lack of standardized methods to assess chemotherapy-induced neurotoxicity. The consensus is that this approach must include objective evidence of neurological deficits and assessment of symptoms from the patient’s perspective, as clinician-based reports of adverse events during chemotherapy often underestimate the severity and frequency of CIPN compared to patient reports (38). Particularly, subjective symptoms like fatigue and numbness significantly impact a patient’s quality of life. Therefore, clinical management and preventive intervention trials necessitate the utilization of refined instruments to gauge CIPN severity. These instruments should meet stringent bioassay criteria, including simplicity, responsiveness, and reproducibility (39).

Clear conclusions about the efficacy of ice gloves remain elusive. Future studies should prioritize large, multicenter randomized controlled trials to clarify the efficacy of ice gloves in the prevention of CIPN, and more studies are needed to demonstrate the maximum efficacy of the correct use of ice gloves when using different chemotherapy regimens and chemotherapeutic drug doses. In addition, the study of the optimal duration and temperature of ice glove application to prevent hypothermia in patients also requires extensive exploration.

## 5 Conclusion

The results of this meta-analysis, combined with previous similar experiments, suggest that frozen gloves can improve the quality of life of breast cancer patients by reducing the incidence and severity of CIPN. However, the effectiveness of ice gloves in preventing CIPN remains inconclusive due to the low quality and limited number of clinical trials on this topic. As a result, more high-quality and well-designed trials are needed for standardized protocols.

## Data availability statement

The original contributions presented in the study are included in the article/**Supplementary Material**. Further inquiries can be directed to the corresponding author.

## Author contributions

HW: Writing – original draft. YJ: Writing – original draft. JS: Writing – original draft. XG: Writing – review & editing.

## References

[1] Sung H Ferlay J Siegel RL Laversanne M Soerjomataram I Jemal A . Global cancer statistics 2020: GLOBOCAN estimates of incidence and mortality worldwide for 36 cancers in 185 countries. CA: Cancer J Clin. (2021) 71:209–49. doi: 10.3322/caac.21660 33538338

[2] Zajączkowska R Kocot-Kępska M Leppert W Wrzosek A Mika J Wordliczek J . Mechanisms of chemotherapy-induced peripheral neuropathy. Int J Mol Sci. (2019) 20(6):1451. doi: 10.3390/ijms20061451 30909387 PMC6471666

[3] Staff NP Grisold A Grisold W Windebank AJ . Chemotherapy-induced peripheral neuropathy: A current review. Ann Neurol. (2017) 81:772–81. doi: 10.1002/ana.24951 PMC5656281 28486769

[4] Argyriou AA Bruna J Marmiroli P Cavaletti G . Chemotherapy-induced peripheral neurotoxicity (CIPN): an update. Crit Rev Oncol Hematol. (2012) 82:51–77. doi: 10.1016/j.critrevonc.2011.04.012 21908200

[5] Stubblefield MD McNeely ML Alfano CM Mayer DK . A prospective surveillance model for physical rehabilitation of women with breast cancer: chemotherapy-induced peripheral neuropathy. Cancer. (2012) 118:2250–60. doi: 10.1002/cncr.27463 22488699

[6] Ma J Kavelaars A Dougherty PM Heijnen CJ . Beyond symptomatic relief for chemotherapy-induced peripheral neuropathy: Targeting the source. Cancer. (2018) 124:2289–98. doi: 10.1002/cncr.31248 PMC5991994 29461625

[7] Mols F Beijers T Lemmens V van den Hurk CJ Vreugdenhil G van de Poll-Franse LV . Chemotherapy-induced neuropathy and its association with quality of life among 2- to 11-year colorectal cancer survivors: results from the population-based PROFILES registry. J Clin Oncol. (2013) 31:2699–707. doi: 10.1200/JCO.2013.49.1514 23775951

[8] Loprinzi CL Lacchetti C Bleeker J Cavaletti G Chauhan C Hertz DL . Prevention and management of chemotherapy-induced peripheral neuropathy in survivors of adult cancers: ASCO guideline update. J Clin Oncol. (2020) 38:3325–48. doi: 10.1200/JCO.20.01399 32663120

[9] Jie ZZ Yun L Cheng SC Sheng PP Wen HZ Juan SF . Research progress on the prevention and treatment of chemotherapy-induced peripheral neuropathy with non-pharmacological therapies. Med Rev. (2019) 25:4909–13 + 18. Available at: https://link.cnki.net/urlid/11.3553.r.20191211.1114.036.

[10] Ruddy KJ Le-Rademacher J Lacouture ME Wilkinson M Onitilo AA Vander Woude AC . Randomized controlled trial of cryotherapy to prevent paclitaxel-induced peripheral neuropathy (RU221511I); an ACCRU trial. Breast (Edinburgh Scotland). (2019) 48:89–97. doi: 10.1016/j.breast.2019.09.011 31590108 PMC7558814

[11] Sundar R Bandla A Tan S Kumarakulasinghe NB Jeyasekharan AD Ow SGW . The role of limb hypothermia in preventing paclitaxel-induced peripheral neuropathy. J Clin Oncol. (2016) 34:e21696-e21696. doi: 10.1200/JCO.2016.34.15_suppl.e21696 PMC5222823 28119855

[12] Bandla A Tan S Kumarakulasinghe NB Huang Y Ang S Magarajah G . Safety and tolerability of cryocompression as a method of enhanced limb hypothermia to reduce taxane-induced peripheral neuropathy. Supportive Care Cancer. (2020) 28:3691–9. doi: 10.1007/s00520-019-05177-2 PMC7316694 31811482

[13] Shimanuki Y Hashimoto H Kawazoe H Uozumi R Udagawa R Watabe D . Preventive effects of self-administered cryotherapy on paclitaxel-induced peripheral neuropathy in patients with early-stage breast cancer: a propensity score analysis. Die Pharmazie. (2021) 76:261–5. doi: 10.1691/ph.2021.1398 34078520

[14] Beijers AJM Bonhof CS Mols F Ophorst J de Vos-Geelen J Jacobs EMG . Multicenter randomized controlled trial to evaluate the efficacy and tolerability of frozen gloves for the prevention of chemotherapy-induced peripheral neuropathy. Ann Oncol: Off J Eur Soc Med Oncol. (2020) 31:131–6. doi: 10.1016/j.annonc.2019.09.006 31912787

[15] Hanai A Ishiguro H Sozu T Tsuda M Yano I Nakagawa T . Effects of cryotherapy on objective and subjective symptoms of paclitaxel-induced neuropathy: prospective self-controlled trial. J Natl Cancer Institute. (2018) 110:141–8. doi: 10.1093/jnci/djx178 PMC6007752 29924336

[16] Loprinzi CL Lustberg MB Hershman DL Ruddy KJ . Chemotherapy-induced peripheral neuropathy: ice, compression, both, or neither? Ann Oncol. (2020) 31:5–6. doi: 10.1016/j.annonc.2019.10.009 31912795

[17] Page MJ McKenzie JE Bossuyt PM Boutron I Hoffmann TC Mulrow CD . The PRISMA 2020 statement: an updated guideline for reporting systematic reviews. BMJ (Clinical Res ed). (2021) 372:n71. doi: 10.1136/bmj.n71 PMC8005924 33782057

[18] Shimozuma K Ohashi Y Takeuchi A Aranishi T Morita S Kuroi K . Feasibility and validity of the Patient Neurotoxicity Questionnaire during taxane chemotherapy in a phase III randomized trial in patients with breast cancer: N-SAS BC 02. Support Care Cancer. (2009) 17:1483–91. doi: 10.1007/s00520-009-0613-7 19330359

[19] Cella D Peterman A Hudgens S Webster K Socinski MA . Measuring the side effects of taxane therapy in oncology: the functional assessment of cancer therapy-taxane (FACT-taxane). Cancer. (2003) 98:822–31. doi: 10.1002/cncr.11578 12910528

[20] Tan AC McCrary JM Park SB Trinh T Goldstein D . Chemotherapy-induced peripheral neuropathy-patient-reported outcomes compared with NCI-CTCAE grade. Support Care Cancer. (2019) 27:4771–7. doi: 10.1007/s00520-019-04781-6 30972648

[21] Higgins JP Altman DG Gøtzsche PC Jüni P Moher D Oxman AD . The Cochrane Collaboration’s tool for assessing the risk of bias in randomized trials. BMJ (Clinical Res ed). (2011) 343:d5928. doi: 10.1136/bmj.d5928 PMC3196245 22008217

[22] Slim K Nini E Forestier D Kwiatkowski F Panis Y Chipponi J . Methodological index for non-randomized studies (minors): development and validation of a new instrument. ANZ J Surg. (2003) 73:712–6. doi: 10.1046/j.1445-2197.2003.02748.x 12956787

[23] Shigematsu H Kimura Y Itagaki T Yasui D . Persistent weekly paclitaxel-induced peripheral neuropathy in early breast cancer patients enrolled in a randomized trial of cryotherapy. Medicine. (2023) 102:e33580. doi: 10.1097/MD.0000000000033580 37083796 PMC10118320

[24] Shigematsu H Hirata T Nishina M Yasui D Ozaki S . Cryotherapy for the prevention of weekly paclitaxel-induced peripheral adverse events in breast cancer patients. Supportive Care Cancer. (2020) 28:5005–11. doi: 10.1007/s00520-020-05345-9 PMC7447649 32036471

[25] Jue MY Shah D Stiles A Nisar T . Impact of cold therapy on paclitaxel-induced peripheral neuropathy and quality of life in patients with breast cancer. Clin J Oncol Nurs. (2022) 26:93–9. doi: 10.1188/22.CJON.93-99 35073298

[26] Ng DQ Tan CJ Soh BC Tan MML Loh SY Tan YE . Impact of cryotherapy on sensory, motor, and autonomic neuropathy in breast cancer patients receiving paclitaxel: a randomized, controlled trial. Front Neurol. (2020) 11. doi: 10.3389/fneur.2020.604688 PMC7793726 33424755

[27] Xu M Wang F Zhu X Hao Z . Efficacy of cryotherapy on chemotherapy-induced peripheral neuropathy in patients with breast cancer: a propensity score-matched study. Ann Med Surg (2012). (2023) 85:2695–703. doi: 10.1097/MS9.0000000000000906 PMC10289575 37363493

[28] Kanbayashi Y Sakaguchi K Ishikawa T Ouchi Y Nakatsukasa K Tabuchi Y . Comparison of the efficacy of cryotherapy and compression therapy for preventing nanoparticle albumin-bound paclitaxel-induced peripheral neuropathy: a prospective self-controlled trial. Breast (Edinburgh Scotland). (2020) 49:219–24. doi: 10.1016/j.breast.2019.12.011 PMC7375545 31901783

[29] Yang T-T Pai H-C Chen C-Y . Effect of cryotherapy on paclitaxel-induced peripheral neuropathy of the hand in female breast cancer patients: A prospective self-controlled study. Int J Nurs Pract. (2023) 29(4):e13094. doi: 10.1016/j.breast.2019.12.011 35971279

[30] Cavaletti G Frigeni B Lanzani F Mattavelli L Susani E Alberti P . Chemotherapy-Induced Peripheral Neurotoxicity assessment: A critical revision of the currently available tools. Eur J Cancer. (2010) 46:479–94. doi: 10.1016/j.ejca.2009.12.008 20045310

[31] Binder J Unver E Clayton J Burke P Paxman R Sundar R . A limb hypothermia wearable for chemotherapy-induced peripheral neuropathy: A mixed-methods approach in medical product development. Front Digital Health. (2020) 2:573234. doi: 10.3389/fdgth.2020.573234 PMC8521967 34713046

[32] Jia J Guo Y Sundar R Bandla A Hao Z . Cryotherapy for prevention of taxane-induced peripheral neuropathy: A meta-analysis. Front Oncol. (2021) 11. doi: 10.3389/fonc.2021.781812 PMC8667340 34912720

[33] Bailey AG Brown JN Hammond JM . Cryotherapy for the prevention of chemotherapy-induced peripheral neuropathy: A systematic review. J Oncol Pharm Pract. (2021) 27:156–64. doi: 10.1177/1078155220959431 32955997

[34] Beijers AJM Bonhof CS Mols F Ophorst J de Vos-Geelen J Jacobs EMG . Multicenter randomized controlled trial to evaluate the efficacy and tolerability of frozen gloves for the prevention of chemotherapy-induced peripheral neuropathy. Ann Oncol. (2020) 31:131–6. doi: 10.1016/j.annonc.2019.09.006 31912787

[35] Beh ST Kuo YM Chang WW Wilder-Smith E Tsao CH Tsai CH . Preventive hypothermia as a neuroprotective strategy for paclitaxel-induced peripheral neuropathy. Pain. (2019) 160:1505–21. doi: 10.1097/j.pain.0000000000001547 30839425

[36] Liao LD Orellana J Liu YH Lin YR Vipin A Thakor NV . Imaging of temperature dependent hemodynamics in the rat sciatic nerve by functional photoacoustic microscopy. Biomed Eng Online. (2013) 12:120. doi: 10.1186/1475-925X-12-120 24245952 PMC4225521

[37] Nilsson AL . Blood flow, temperature, and heat loss of skin exposed to local radiative and convective cooling. J Invest Dermatol. (1987) 88:586–93. doi: 10.1111/1523-1747.ep12470202 3553342

[38] Park SB Goldstein D Krishnan AV Lin CSY Friedlander ML Cassidy J . Chemotherapy-induced peripheral neurotoxicity: A critical analysis. CA Cancer J Clin. (2013) 63:419–37. doi: 10.3322/caac.21204 24590861

[39] Argyriou A Boogerd W Briani C Bruna J Faber CG Grisold W . CI-PERINOMS: chemotherapy-induced peripheral neuropathy outcome measures study. J Peripheral Nervous Syst: JPNS. (2009) 14:69–71. doi: 10.1111/j.1529-8027.2009.00214.x 19691527

