# Supplementary material for: Effect of frozen gloves on chemotherapy-induced neurotoxicity in breast cancer patients: a systematic review and meta-analysis
Source: Front Oncol. 2024 Jun 6;14:1366782. doi: 10.3389/fonc.2024.1366782 (PMC11187580; doi:10.3389/fonc.2024.1366782)
Supplement: Supplementary file 1 [file DataSheet_1.docx]

Supplementary Material

**Search strategy： n=320**

1. **Web of Science n=44**

#1. **(((((TS=("breast neoplasms" )) OR TS=("breast neoplasm")) OR TS=( "breast tumor")) OR TS=("breast cancer" )) OR TS=( "mammary cancer" )) OR TS=("breast carcinoma" )**

**#2. (((((((((TS=("Peripheral Nervous System Disease")) OR TS=( "chemotherapy induced peripheral neuropathy" )) OR TS=("peripheral neuropathy")) OR TS=("peripheral neurotoxicity" )) OR TS=(CIPN)) OR TS=( "chemotherapy induced neurotoxicity" )) OR TS=(neuralgia)) OR TS=( paresthesia )) OR TS=(neurotoxicity)) OR TS=(neuropathy)**

**#3. (((((((((TS=(cryotherapy)) OR TS=( "Cryotherapy therapy" )) OR TS=( "cold therapy" )) OR TS=(hypothermia)) OR TS=( "low temperature")) OR TS=(cryotherapies )) OR TS=("cold therapies")) OR TS=(ice)) OR TS=(cryogenic)) OR TS=("frozen gloves")**

**#4. #3 AND #2 AND #1**

1. **PubMed n=22**

#1. "Breast Neoplasms"[MeSH Terms]

#2. "breast neoplasm"[Title/Abstract] OR "breast tumor"[Title/Abstract] OR "breast cancer"[Title/Abstract] OR "mammary cancer"[Title/Abstract] OR "breast carcinoma"[Title/Abstract]

#3. "Peripheral Nervous System Diseases"[MeSH Terms]

#4. "chemotherapy induced peripheral neuropathy"[Title/Abstract] OR "peripheral neuropathy"[Title/Abstract] OR "peripheral neurotoxicity"[Title/Abstract] OR "CIPN"[Title/Abstract] OR "chemotherapy induced neurotoxicity"[Title/Abstract] OR "neuralgia"[Title/Abstract] OR "paresthesia"[Title/Abstract] OR "neurotoxicity"[Title/Abstract] OR "neuropathy"[Title/Abstract]

#5. "Cryotherapy"[MeSH Terms]

#6. "cold therapy"[Title/Abstract] OR "hypothermia"[Title/Abstract] OR "low temperature"[Title/Abstract] OR "cryotherapies"[Title/Abstract] OR "cold therapies"[Title/Abstract] OR "ice"[Title/Abstract] OR "cryogenic"[Title/Abstract] OR "frozen gloves"[Title/Abstract]

#7. #1 OR #2

#8. #3 OR #4

#9. #5 OR #6

#10. #7 AND #8 AND #9

1. **Embase n=79**

#1. 'breast tumor'/exp

#2. 'breast neoplasms':ti,ab,kw OR 'breast neoplasm':ti,ab,kw OR 'breast cancer':ti,ab,kw OR 'mammary cancer':ti,ab,kw OR 'breast carcinoma':ti,ab,kw

#3. 'peripheral neuropathy'/exp

#4. 'peripheral nervous system disease':ti,ab,kw OR 'chemotherapy induced peripheral neuropathy':ti,ab,kw OR 'peripheral neurotoxicity':ti,ab,kw OR cipn:ti,ab,kw OR 'chemotherapy induced neurotoxicity':ti,ab,kw OR neuralgia:ti,ab,kw OR paresthesia:ti,ab,kw OR neurotoxicity:ti,ab,kw OR neuropathy:ti,ab,kw

#5. 'cryotherapy'/exp

#6. 'cryotherapy therapy':ti,ab,kw OR 'cold therapy':ti,ab,kw OR hypothermia:ti,ab,kw OR 'low temperature':ti,ab,kw OR cryotherapies:ti,ab,kw OR 'cold therapies':ti,ab,kw OR ice:ti,ab,kw OR cryogenic:ti,ab,kw OR 'frozen gloves':ti,ab,kw

#7. #1 OR #2

#8. #3 OR #4

#9. #5 OR #6

#10. #7 AND #8 AND #9

1. **Cochrane Library n=41**

#1. MeSH descriptor: [Breast Neoplasms] explode all trees

#2. ("breast neoplasm"):ti,ab,kw OR ("breast tumor"):ti,ab,kw OR ("breast cancer"):ti,ab,kw OR ("mammary cancer"):ti,ab,kw OR ("breast carcinoma"):ti,ab,kw

#3. MeSH descriptor: [Peripheral Nervous System Diseases] explode all trees

#4. ("chemotherapy induced peripheral neuropathy"):ti,ab,kw OR ("peripheral neuropathy"):ti,ab,kw OR ("peripheral neurotoxicity"):ti,ab,kw OR (CIPN):ti,ab,kw OR ("chemotherapy induced neurotoxicity"):ti,ab,kw

#5. (neuralgia):ti,ab,kw OR (paresthesia):ti,ab,kw OR (neurotoxicity):ti,ab,kw OR (neuropathy):ti,ab,kw

#6. MeSH descriptor: [Cryotherapy] explode all trees

#7. ("Cryotherapy therapy"):ti,ab,kw OR ("cold therapy"):ti,ab,kw OR (hypothermia):ti,ab,kw OR ("low temperature"):ti,ab,kw OR (cryotherapies):ti,ab,kw

#8. ("cold therapies"):ti,ab,kw OR (ice):ti,ab,kw OR (cryogenic):ti,ab,kw OR ("frozen gloves"):ti,ab,kw

#9 #1 OR #2

#10 #3 OR #4 OR #5

#11 #6 OR #7 OR #8

#12 #9 AND #10 AND #11

1. **Scopus n=79**

#1. ( TITLE-ABS-KEY ( "breast neoplasms" ) OR TITLE-ABS-KEY ( "breast neoplasm" ) OR TITLE-ABS-KEY ( "breast tumor" ) OR TITLE-ABS-KEY ( "breast cancer" ) OR TITLE-ABS-KEY ( "mammary cancer" ) OR TITLE-ABS-KEY ( "breast carcinoma" ) )

#2. ( TITLE-ABS-KEY ( "peripheral nervous system disease" ) OR TITLE-ABS-KEY ( "chemotherapy induced peripheral neuropathy" ) OR TITLE-ABS-KEY ( "peripheral neuropathy" ) OR TITLE-ABS-KEY ( "peripheral neurotoxicity" ) OR TITLE-ABS-KEY ( cipn ) OR TITLE-ABS-KEY ( "chemotherapy induced neurotoxicity" ) OR TITLE-ABS-KEY ( neuralgia ) OR TITLE-ABS-KEY ( paresthesia ) OR TITLE-ABS-KEY ( neurotoxicity ) OR TITLE-ABS-KEY ( neuropathy ) )

#3. ( TITLE-ABS-KEY ( cryotherapy ) OR TITLE-ABS-KEY ( "cryotherapy therapy" ) OR TITLE-ABS-KEY ( "cold therapy" ) OR TITLE-ABS-KEY ( hypothermia ) OR TITLE-ABS-KEY ( "low temperature" ) OR TITLE-ABS-KEY ( cryotherapies ) OR TITLE-ABS-KEY ( "cold therapies" ) OR TITLE-ABS-KEY ( ice ) OR TITLE-ABS-KEY ( cryogenic ) OR TITLE-ABS-KEY ( "frozen gloves" ) )

**#4. #3 AND #2 AND #1**

1. **EBSCO n=18**

#1. AB "breast neoplasms" OR AB "breast neoplasm" OR AB "breast tumor" OR AB "breast cancer" OR AB "mammary cancer" OR AB "breast carcinoma"

#2. AB "Peripheral Nervous System Disease" OR AB "chemotherapy induced peripheral neuropathy" OR AB "peripheral neuropathy" OR AB "peripheral neurotoxicity" OR AB CIPN OR AB "chemotherapy induced neurotoxicity" OR AB neuralgia OR AB paresthesia OR AB neurotoxicity OR AB neuropathy

#3. AB cryotherapy OR AB "Cryotherapy therapy" OR AB "cold therapy" OR AB hypothermia OR AB "low temperature" OR AB cryotherapies OR AB "cold therapies" OR AB ice OR AB cryogenic OR AB "frozen gloves"

#4. #1 AND #2 AND #3

1. **OVID n=37**

#1. ("breast neoplasms" or "breast neoplasm" or "breast tumor" or "breast cancer" or "mammary cancer" or "breast carcinoma").ab,kw,ti.

#2. ("Peripheral Nervous System Disease" or "chemotherapy induced peripheral neuropathy" or "peripheral neuropathy" or "peripheral neurotoxicity" or CIPN or "chemotherapy induced neurotoxicity" or neuralgia or paresthesia or neurotoxicity or neuropathy).ab,kw,ti.

#3. (cryotherapy or "Cryotherapy therapy" or "cold therapy" or hypothermia or "low temperature" or cryotherapies or "cold therapies" or ice or cryogenic or "frozen gloves").ab,kw,ti.

#4. #1 AND #2 AND #3

1. **CNKI n=0**

Search query= (SU= ‘乳腺癌’ OR SU= ‘乳房恶性肿瘤’ OR SU= ‘乳岩’) AND (SU= ‘化疗所致周围神经毒性’ OR SU= ‘周围神经病变’ OR SU= ‘化疗相关性周围神经病变’ OR SU= ‘外周神经毒性’ OR SU= ‘外周神经病变’ ) AND (SU= ‘冷冻疗法’ OR SU= ‘冰手套’ OR SU= ‘冰冻手套’ ）

1. **WangFang n=1**

Search query= 主题: (“乳腺癌” OR “乳房恶性肿瘤” OR “乳岩”) and 主题:(“化疗所致周围神经毒性” OR “周围神经病变” OR “化疗相关性周围神经病变” OR “外周神经毒性” OR “外周神经病变”) and 主题:(“冷冻疗法” OR “冰手套” OR “冰冻手套”)
